# Authorship issue explained

**DOI:** 10.4103/0970-0358.73482

**Published:** 2010

**Authors:** Surajit Bhattacharya

**Affiliations:** Editor, IJPS E-mail: surajitbh@yahoo.co.in

When it comes to the fact that who should be an author and who should not be offered ghost authorship, it seem we are all in agreement.[1] Each author should have participated sufficiently in the work to take responsibility for the content. Authorship credit should be based only on substantial contributions to (a) conception and design, or analysis and interpretation of data; and to (b) drafting the article or revising it critically for important intellectual content; and on (c) final revision of the version to be published. Conditions (a), (b), and (c) must all are met.

However, when it comes to the sequence of authorship there seems to be a grey zone and exploitation at both ends of the spectrum. We have come across aggrieved Unit Chiefs and displeased residents in almost equal numbers. It is important for young authors to understand that there are two positions that count, the first author and the last author. Attached to either position is the status associated with being the author for correspondence. The best combination when one is young is to be first author and the author for correspondence. As one’s career progresses, being last author and author for correspondence signals that this is a paper from one’s Unit, he/she is the main person responsible for its contents, and a younger colleague has made major contributions to the paper, hence he/she is designated as the first author. The guidelines here are not as well defined as for authorship in general, Riesenberg and Lundberg[2] have made certain very important and simple suggestions to decide the sequence of authorship:

1. The first author should be that person who contributed most to the work, including writing of the manuscript
2. The sequence of authors should be determined by the relative overall contributions to the manuscript.
3. It is common practice to have the senior author appear last, sometimes regardless of his or her contribution. The senior author, like all other authors, should meet all criteria for authorship.
4. The senior author sometimes takes responsibility for writing the paper, especially when the research student has not yet learned the skills of scientific writing. The senior author then becomes the corresponding author, but should the student be the first author? Some supervisors put their students first, others put their own names first. Perhaps it should be decided on the absolute amount of time spent on the project by the student (in getting the data) and the supervisor (in providing help and in writing the paper). Or perhaps the supervisor should be satisfied with being corresponding author, regardless of time committed to the project.
5. A sensible policy adopted by many supervisors is to give the student a fixed period of time (say 12 months) to write the first draft of the paper. If the student does not deliver, the supervisor may then write the paper and put her or his own name first.

The second issue raised in this letter is about the use of plurals. Our insistence of avoiding pronouns I, me and mine in all publications is very sound and logical. Even if it is a single author paper, surgery is a team game and we are virtually powerless without our unsung colleagues - residents, nurses, technicians etc. By using plurals we recognize their vital role in our success story. Where as in a multiple author paper, the author has no option but to call it ‘our work’ instead on ‘my paper’, even when he is writing the paper all by himself / herself, there were many hands helping him / her and it is our Journal policy to acknowledge the same.

## References

[1] (1991). International Committee for Medical Journal Editors. Uniform requirements for manuscripts submitted to biomedical journals. N Engl J Med.

[2] Riesenberg D, Lundberg GD (1990). The order of authorship: who’s on first?. JAMA.

[3] Relman AS (1984). Responsibilities for authorship: where does the buck stop?. N Engl J Med.

[4] Huth EJ (1986). Guidelines on authorship of medical papers. Ann Intern Med.

[5] Mohta A (2010). Authorship issues continued. Indian J Plast Surg.

